# Targeting SIRT3 to Ameliorate Diabetic Cardiomyopathy: Progress in Mechanistic Research and Prospects for Clinical Translation

**DOI:** 10.1111/1753-0407.70167

**Published:** 2025-11-23

**Authors:** Yuting Lin, Kun Yu, Jinjian Guo

**Affiliations:** ^1^ The Second People's Hospital Affiliated to Fujian University of Traditional Chinese Medicine Fuzhou Fujian China

**Keywords:** cardiomyocyte death, diabetic cardiomyopathy, mitochondrial dysfunction, SIRT3, therapy

## Abstract

Diabetes mellitus (DM) is one of the most common chronic diseases worldwide, and diabetic cardiomyopathy (DCM) is one of the cardiovascular complications of DM, described as the development of abnormalities of myocardial structure/function associated with DM in the absence of coronary artery disease, hypertension, and valvular disease. The disease has an insidious onset and lacks effective treatment. Studies have shown that even with effective glycemic control, the progression of DCM cannot be prevented. Exploring the pathogenesis of DCM and identifying effective intervention targets is the focus and hotspot of current research. Silent message regulator 3 (Sirtuin3, SIRT3) is one of the members of the nicotinamide adenine dinucleotide (NAD^+^)‐dependent deacetylase family of sirtuins, and studies have confirmed that SIRT3 has a protective effect on cardiovascular disease and may become a new target for the treatment of cardiovascular diseases. Therefore, this paper emphasizes the role of SIRT3 in DCM, describes the mechanism of SIRT3 in DCM, and summarizes the methods to improve DCM by elevating the level of SIRT3, aiming to provide new perspectives for treating and delaying DCM.

## Graphical abstract

Diabetic cardiomyopathy is one of the cardiovascular complications of diabetes, and currently lacks corresponding treatment options. This review emphasizes the mechanism of action of SIRT3 in improving DCM and summarizes methods for improving DCM by increasing SIRT3 levels.

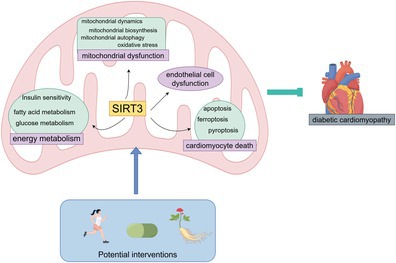

**Summary**

- First comprehensive integration of SIRT3's multi‐dimensional protective roles in DCM, spanning energy metabolism, mitochondrial quality control, cardiomyocyte death suppression, and endothelial repair.
- A new perspective on breaking through the treatment dilemma of “high blood sugar memory”. It is proposed that SIRT3 agonists may bypass the limitations of traditional hypoglycemic therapy, providing a new strategy to overcome the “metabolic memory” of diabetic myocardium.
- Translational bridge from basic to clinical. Summarizes the types of interventions targeting SIRT3, including lifestyle modifications, Western drugs, and natural compounds, to provide a roadmap for drug development.

## 1 Introduction

Diabetes mellitus (DM) is one of the major diseases threatening human health, with an increasing global prevalence that is expected to reach 784 million by 2045 [1]. DM causes a variety of serious complications involving vital organs such as the heart, kidneys, and eyes, with cardiovascular complications having the highest prevalence and mortality rates. Diabetic cardiomyopathy (DCM) is one of the cardiovascular complications of DM, which is characterized by abnormalities in myocardial structure and function, and is different from myocardial injury caused by coronary artery ischemia, hypertension, and valvular disease [2]. The onset of DCM is insidious, and the disease is easily detected only when it has progressed to heart failure. At present, there are no specific treatments for the disease, which is mainly based on symptomatic management. However, several large‐scale studies have shown that diabetic patients have “hyperglycemic memory”, and even with intensive glycemic control, the progression of DCM cannot be delayed [3, 4]. Therefore, many researchers are committed to exploring the pathogenesis of DCM and finding effective targets for intervention.

Silent information regulator 3 (Sirtuin3, SIRT3) is a member of the nicotinamide adenine dinucleotide (NAD^+^)‐dependent deacetylase family of sirtuins, which is mainly found in mitochondria and has an important role in the regulation of mitochondrial homeostasis [5, 6]. Studies have shown that SIRT3 has cardioprotective effects and can effectively reduce myocardial hypertrophy, fibrosis, and injury [7]. Previous experimental studies have shown that overexpression of SIRT3 leads to reduced reactive oxygen species (ROS) generation and improved cardiac function in a diabetic mouse model [8]. In STZ‐induced DCM mice, SIRT3 knockout (SIRT3 KO) inhibits mitochondrial autophagy, causing mitochondrial damage, cardiomyocyte apoptosis, and interstitial fibrosis, leading to cardiac dysfunction in DCM [9]. In conclusion, these studies have demonstrated the important role of SIRT3 in cardiovascular disease and may be a new target for the treatment of cardiovascular disease, but the specific mechanism by which SIRT3 ameliorates myocardial tissue injury in DCM patients remains to be refined. Therefore, this paper reviews the research progress on the role of SIRT3 in DCM, aiming to provide the theoretical basis and ideas for the study of DCM.

## 2 SIRT3 Structure and Function

**FIGURE 1:**
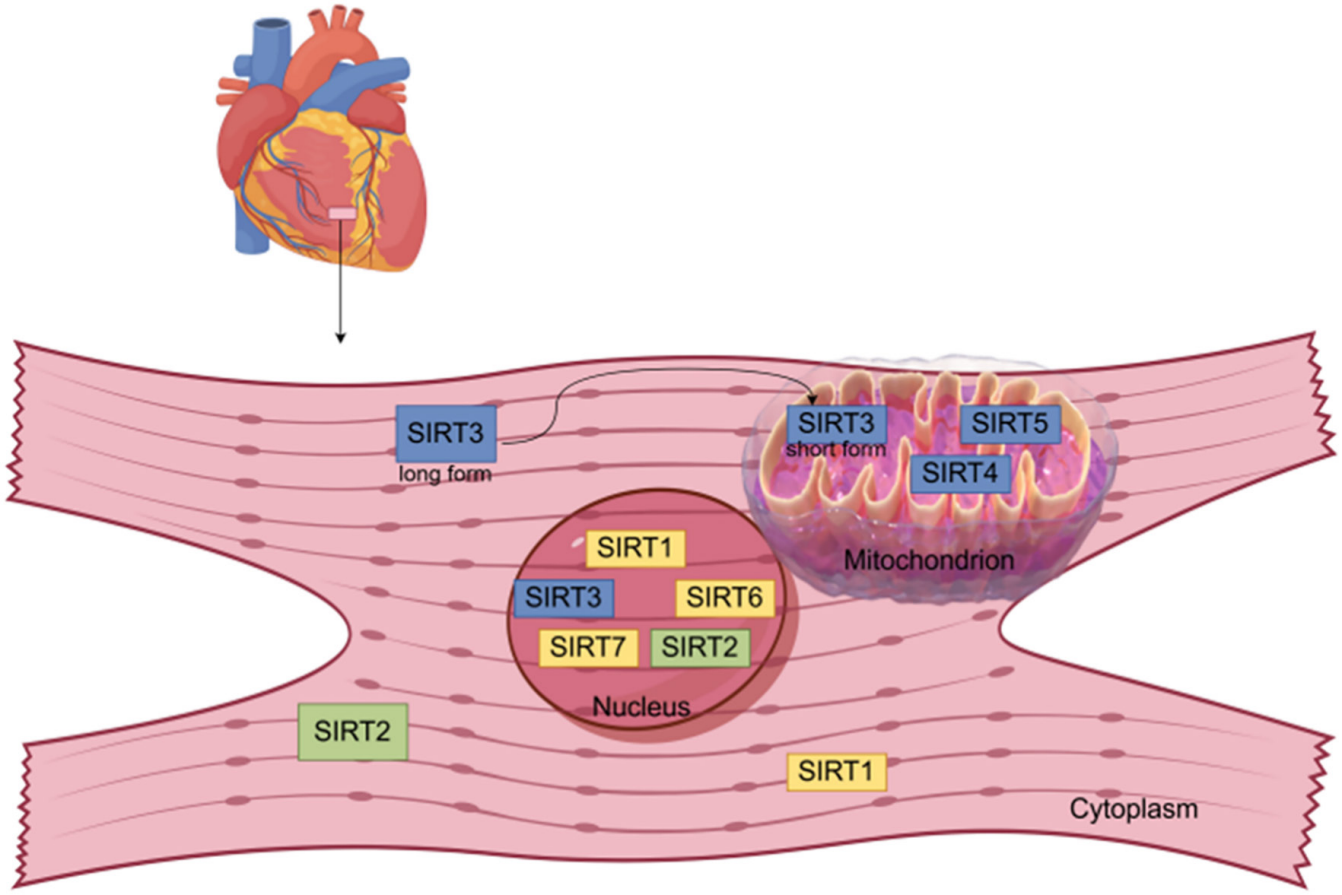
Subcellular localization of SIRT1–7. SIRT1, silent information regulator 1; SIRT2, silent information regulator 2; SIRT3, silent information regulator 3; SIRT4, silent information regulator 4; SIRT5, silent information regulator 5; SIRT6, silent information regulator 6; SIRT7, silent information regulator 7.

The SIRT family is NAD^+^‐dependent protein deacetylases that contain seven members (SIRT1–7) in mammals [10]. The members of the SIRT family are distributed in different organelles (Figure 1). SIRT1, SIRT6, and SIRT7 are mainly distributed in the nucleus, SIRT2 is predominantly in the cytoplasm, SIRT3, SIRT4, and SIRT5 are mainly found in mitochondria and are involved in various regulatory processes such as gene transcription and cellular metabolism, etc., respectively. In recent years, it has been found that SIRT3 is differentially expressed in the cytoplasm and mitochondria [11]. This may be due to differences in SIRT3 isoforms (full‐length and short form) [12]. When cells are stressed, full‐length SIRT3 is transferred from the cytoplasm to the mitochondria and cleaved to the short form by matrix processing peptidase (MPP) [13]. Initially, full‐length SIRT3 was considered an inert enzyme without enzymatic activity. Subsequent studies revealed that both SIRT3 isoforms possess deacetylase activity [14], suggesting that SIRT3's functional role may not be constrained by its form or location. Current understanding of SIRT3's subcellular localization and function remains inconclusive. Most studies suggest that SIRT3 is primarily localized to mitochondria, where it exhibits potent deacetylase activity and primarily regulates mitochondrial function. Therefore, further research is needed to determine the precise subcellular localization of SIRT3 and its corresponding functions.

In terms of molecular structure, the core enzyme region of SIRT3 consists of two key structural domains: a larger rossmann‐folded structural domain responsible for binding NAD^+^, and a smaller structural domain consisting of a helical complex structure and a zinc‐binding motif. The gap formed between these two structural domains is the main binding site for acetylated substrates [14]. It is noteworthy that the expression level of SIRT3 changes with age, and this change manifests itself differently in different tissues. For example, in tissues such as the lung and spleen, the expression of SIRT3 is up‐regulated with age; whereas in the kidney, adipose tissue, skin, and myocardium, it shows a down‐regulation trend. This differential expression pattern may be an adaptive regulatory mechanism adopted by the body to maintain the functional homeostasis of each tissue [15].

As a key deacetylase, SIRT3 possesses potent deacylation activity, which can directly participate in regulating the acetylation state of at least 84 mitochondrial proteins, which encompass virtually all mitochondrial biological functions, including mitochondrial mitosis, transcription, translation, DNA processing, RNA processing, the tricarboxylic acid cycle, and amino acid metabolism [16]. SIRT3 exerts its biological functions by regulating metabolism, inhibiting oxidative stress, and delaying cellular senescence [17, 18]. These functions have enabled it to exhibit significant potential in the prevention and treatment of a variety of diseases, including metabolic syndrome, neurodegenerative diseases, and cardiovascular diseases [19, 20, 21]. Notably, recent studies have revealed the unique regulatory mechanism of SIRT3 in the cardiovascular system: by precisely regulating myocardial metabolic processes and modulating mitochondrial homeostasis to alleviate myocardial hypertrophy and fibrosis, it shows breakthrough applications in the treatment of cardiovascular diseases [18]. This innovative discovery not only deepens the understanding of the function of SIRT3 but also provides new clues for targeted therapy of cardiovascular diseases.

## 3 SIRT3 and DCM

**FIGURE 2:**
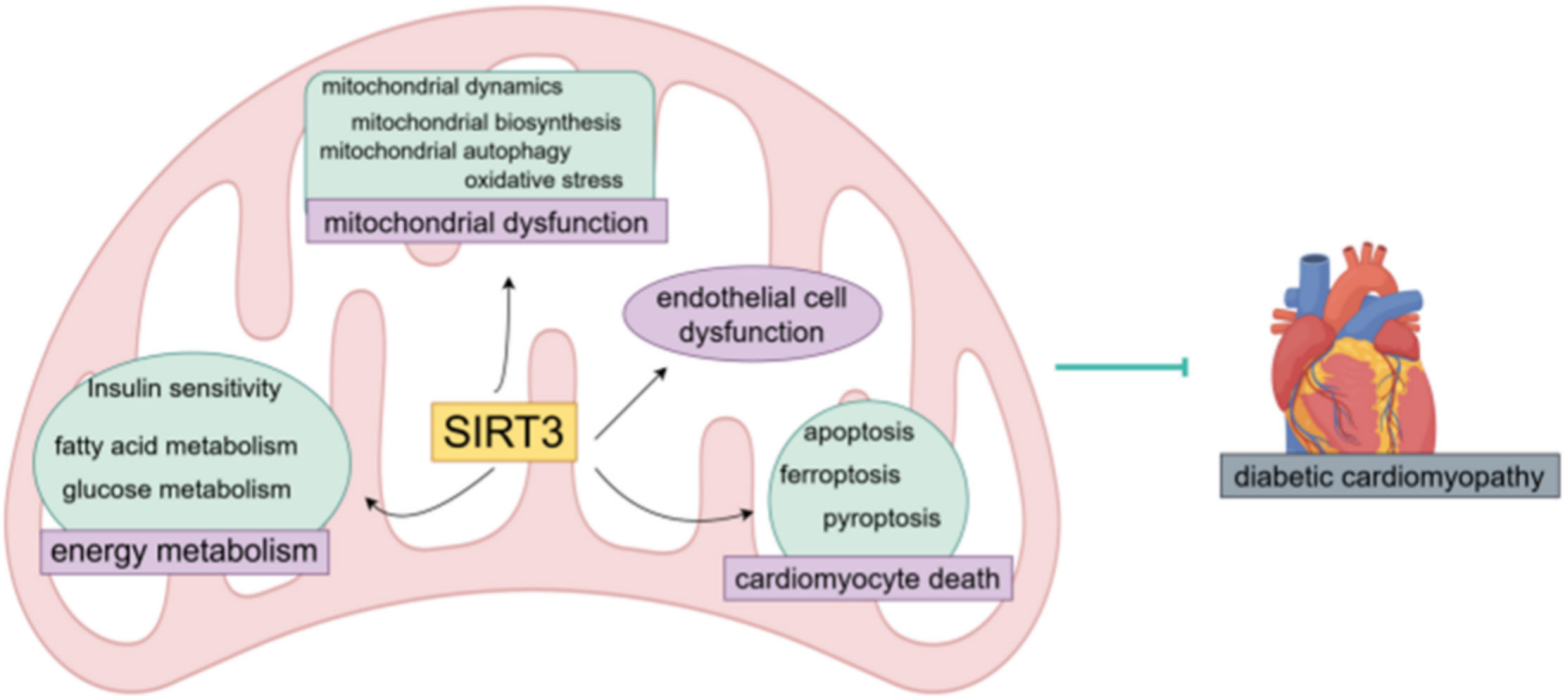
The regulatory role of SIRT3 in DCM. SIRT3, silent information regulator 3.

DCM was first defined by Rubler in 1972 as the development of left ventricular remodeling in diabetic patients in the absence of coronary artery disease, hypertension, and valvular heart disease, ultimately leading to cardiac systolic and/or diastolic dysfunction [22]. DM‐associated disorders of glucose metabolism cause DCM, which is characterized by major pathological features such as structural abnormalities in cardiomyocytes and local microvascular dysfunction. Although the exact pathophysiological mechanisms of DCM are not known, oxidative stress, mitochondrial dysfunction, endoplasmic reticulum stress, inflammation, autophagy, myocardial fibrosis, lipotoxicity, and cardiomyocyte death have been found to be associated with it. It is noteworthy that SIRT3, as a key regulatory molecule, plays a significant role in maintaining myocardial homeostasis in DCM by regulating energy metabolism, improving mitochondrial dysfunction, inhibiting cardiomyocyte death, and enhancing endothelial cell function (Figure 2). The following section will comprehensively elaborate on the regulatory role of SIRT3 in DCM.

### 3.1 SIRT3 and Energy Metabolism in the DCM

Myocardium is one of the human tissues with highly active metabolic activities, and glucose is the most efficient fuel for the heart. Cardiomyocytes take up glucose through glucose transporter proteins 1 and 4 (GLUT1 and GLUT4), and glycolysis occurs in the cytoplasm to generate pyruvic acid, which then enters the mitochondria to participate in the tricarboxylic acid cycle and produce a large amount of ATP. However, chronic hyperglycemia impairs the insulin signaling of the myocardial cells, leading to cardiac insulin resistance, and impairs glucose uptake by cardiomyocytes. Studies have shown that SIRT3 ameliorates insulin resistance in cardiomyocytes of obese mice by up‐regulating AMP‐activated protein kinase (AMPK), and down‐regulating hypoxia‐inducible factor‐1α (HIF‐1α), deacetylating peroxisome proliferator‐activated receptor‐γ coactivator‐1α (PGC‐1α) and pyruvate dehydrogenase (PDH) [23, 24]. Forkhead box class O proteins (FOXOs) are important transcription factors in cardiomyocytes, and SIRTs regulate the transcriptional activity of FOXOs mainly through acetylation/deacetylation, thus playing a role in a variety of pathophysiological processes [25]. Animal experiments have shown that SIRT3 can improve cellular insulin‐stimulated glucose uptake by deacetylating FOXO3 [26]. In addition, decreased SIRT3 expression in cardiomyocytes under high glucose conditions leads to increased tumor protein p53 (p53) acetylation, which in turn upregulates the expression of its downstream target gene TP53‐induced glycolysis and apoptosis regulator (TIGAR), a process that is significantly inhibited by adenovirus‐mediated SIRT3 overexpression [8]. Thus, SIRT3 plays an important role in insulin sensitivity and glucose metabolism in cardiomyocytes.

Under physiological conditions, fatty acid (FA) oxidation provides most of the energy (60%–90%) for mitochondrial oxidative phosphorylation in cardiomyocytes, with glucose metabolism and lactate metabolism accounting for the rest [27, 28, 29]. It has been shown that SIRT3 regulates fatty acid oxidation through deacetylation and modification of several key enzymes [30]. Long‐chain acyl‐CoA dehydrogenase (LCAD), a key enzyme in fatty acid oxidation, catalyzes the first step in fatty acid β‐oxidation, and SIRT3‐KO mice were found to have higher LCAD acetylation and impaired fatty acid oxidation [31]. Activation of SIRT3, which mediated LCAD deacetylation, increased fatty acid oxidation and improved metabolic remodeling in patients with atrial fibrillation [32]. In DCM, glucose utilization is further reduced due to insulin resistance, forcing the heart to rely more on fatty acid oxidation. Fatty acid oxidation, however, requires the consumption of large amounts of oxygen, and excessive fatty acid oxidation, which causes insufficient oxygen supply to the heart, diverts certain fatty acids to non‐oxidative pathways, generating toxic lipids, such as ceramides and diacylglycerol, which cause impairment of mitochondrial function [33, 34]. Experimental studies have shown that fatty acid oxidation is impaired and metabolism is significantly abnormal in the myocardium of mice with SIRT3‐KO [35], and the expression of both GLUT1 and GLUT4 is impaired, which in turn leads to the obstruction of the glucose transport process to the cardiomyocytes, and the inability of cardiomyocytes to obtain sufficient glucose, which then affects cardiac energy metabolism [36, 37]. Sun et al. [38] found that exogenous hydrogen sulfide modulated the expression of SIRT3 in db/db mice, which induced a shift in cardiac energy substrate utilization from fatty acid β‐oxidation to glucose oxidation in DCM, thereby ameliorating myocardial fibrosis in DCM. In conclusion, SIRT3 is important for fatty acid metabolism and glucose metabolism in DCM.

### 3.2 SIRT3 And Mitochondrial Dysfunction in DCM

Organisms need to consume large amounts of ATP every day for their own energy homeostasis, with the majority (> 95%) coming from oxidative phosphorylation in mitochondria. Although the human heart is only 0.5% of body mass, it consumes about 8% of ATP [39, 40]. The heart has the highest mitochondrial content compared to other organs [39]. When mitochondrial function is impaired, ATP production is severely affected, leading to cardiac systolic dysfunction [41]. SIRT3 deficiency is associated with cardiac mitochondrial dysfunction, and overexpression of SIRT3 has been shown to ameliorate mitochondrial dysfunction and protect cardiomyocytes [42]. SIRT3 plays a central role in maintaining mitochondrial function through multiple mechanisms.

#### 3.2.1 SIRT3 and Mitochondria‐Associated Oxidative Stress in DCM

Mitochondrial‐mediated oxidative stress is one of the important mechanisms underlying the occurrence and development of DCM. While healthy hearts typically produce small amounts of ROS, increased ROS generation or reduced clearance under abnormal conditions can elevate ROS levels in myocardial tissue. The ameliorative effect of SIRT3 in DCM primarily stems from its ROS scavenging activity. Manganese Superoxide Dismutase (MnSOD), as one of the most important deacetylation substrates of SIRT3, plays a crucial role in ROS clearance. Sultana et al. [43] observed elevated ROS levels and reduced catalase and superoxide dismutase (SOD) activity in the hearts of STZ‐induced type 1 diabetic rats. Garlic administration activated SIRT3, which bound to MnSOD, deacetylated and activated it, thereby scavenging ROS and protecting mice from oxidative stress. Furthermore, hyperglycemia‐induced ROS overproduction prolongs the duration of mitochondrial permeability transition pore (mPTP) opening, thereby reducing mitochondrial membrane potential and impairing ATP synthesis [44]. SIRT3 not only functions as an effective radical scavenger with antioxidant potential but also deacetylates cyclophilin D (CypD) to reduce mPTP opening and improve impaired mitochondrial function [45].

#### 3.2.2 SIRT3 and Mitochondrial Quality Control in DCM

Mitochondrial dynamics refers to the cyclic process in which mitochondria undergo constant fusion and fission, which helps to regulate mitochondrial homeostasis and biosynthesis to maintain energy production and ROS homeostasis. Mitochondrial fusion is mediated by mitochondrial fusion proteins 1 or 2 (Mfn1, Mfn 2) and optic atrophy factor 1 (Opa1); whereas fission is mainly associated with dynamin‐related protein 1 (Drp1), mitochondrial fission protein 1 (FIS1), mitochondrial fission factor (MFF), and mitochondrial fission process 1 (MTFP1) [46]. In hyperglycemia‐induced H9c2 cells, mitochondria can be induced to divide via Drp1 signaling, which leads to the overproduction of ROS [47]; similarly, injecting Mfn2 overexpressing adenovirus into the hearts of db/db mice inhibited mitochondrial fission and delayed the progression of DCM [48]. Ni et al. [49] found that icariin increased the protein expression of PGC‐1α, Mfn2, and Cyt‐b through the Apelin/SIRT3 pathway, improved mitochondrial function, and reduced cardiac fibrosis.

Increasing evidence indicates that dysregulated mitochondrial autophagy exacerbates DCM progression, and maintaining balanced mitochondrial autophagy is crucial for sustaining cellular metabolism in DCM [50, 51]. Studies reveal that resveratrol can modulate mitochondrial autophagy via the AMPK/SIRT1‐mediated IRE1α/PINK signaling pathway, thereby delaying myocardial fibrosis in DCM [52]. Melatonin protects against DCM by upregulating mitochondrial autophagy through MST1/SIRT3 signaling, thereby regulating mitochondrial integrity and biogenesis [53, 54]. These findings collectively demonstrate that activating SIRT3‐related pathways to modulate mitochondrial autophagy contributes to improving DCM outcomes.

### 3.3 SIRT3 and Cardiomyocyte Death in DCM

The pathogenesis of DCM includes disorders of glycolipid metabolism, oxidative stress, inflammatory response, and mitochondrial dysfunction, all of which culminate in cardiomyocyte death. Cardiomyocyte death is the basic pathological process of DCM; therefore, reducing cardiomyocyte death is the key to improving and restoring cardiac function in DCM.

#### 3.3.1 SIRT3 and Apoptosis in DCM

Hyperglycemia, oxidative stress, mitochondrial dysfunction, and inflammation all cause cardiomyocyte apoptosis, which in turn leads to myocardial fibrosis and cardiac dysfunction. It has been found that SIRT3 increases the expression of apoptosis‐related proteins in DCM cardiomyocytes [53], and activation of the SIRT3 pathway inhibits cardiomyocyte apoptosis and improves cardiac function in DCM [55, 56, 57]. Li et al. [8] found that overexpression of SIRT3 significantly improved cardiac function in db/db mice, inhibited the acetylation level of the transcription factor P53 and its downstream target gene TIGAR expression, reduced ROS levels, and inhibited cardiomyocyte apoptosis. Regulation of the FOXO3/Mst1/Sirt3/AMPK axis inhibited cardiomyocyte apoptosis, enhanced mitochondrial autophagy, and ameliorated myocardial fibrosis and cardiac insufficiency in DCM [54]. Dihydromyricetin ameliorated myocardial hypertrophy and fibrosis in DCM through activation of SIRT3, inhibition of oxidative stress, inflammation, and apoptosis, demonstrating its medical potential in DCM treatment [58]. In addition, Zhang et al. [45] found that LncDACH1 directly binds to SIRT3 and promotes its degradation through ubiquitination, thereby promoting mitochondrial oxidative damage and apoptosis in the hearts of DCM mice, whereas SIRT3 silencing abrogated the protective effect of lncDACH1 deficiency on cardiomyocytes.

#### 3.3.2 SIRT3 and Pyroptosis in DCM

Cellular pyroptosis is a newly identified inflammation‐related programmed cell death characterized by cell swelling, plasma membrane rupture, and release of inflammatory cellular contents. Nucleotide oligomerization domain like receptor protein 3 (NLRP3) inflammasome‐mediated cysteinyl asparagin‐1 (Caspase‐1)‐dependent pyroptosis is a typical pathway. NLRP3 inflammasome‐mediated cardiomyocyte pyroptosis is a key player in DCM [59, 60]. It was demonstrated that in human ventricular myocytes, high glucose induced a significant increase in NLRP3, Caspase‐1, and IL‐1β protein expression, which was accompanied by pronounced cardiomyocyte pyroptosis [61]. Similarly, in STZ‐induced diabetic model rats and high glucose‐induced H9C2 cells, NLRP3 and IL‐1β expression were significantly increased compared to controls, whereas activation of the NLRP3 pathway modulated cardiomyocyte pyroptosis and improved myocardial fibrosis and cardiac function in DCM [62]. These findings confirm the unique role of NLRP3 inflammasome‐mediated cellular pyroptosis in DCM. And SIRT3‐mediated NLRP3 inflammasome plays an important role in DCM. Song et al. [53] found that the expression of SIRT3 was decreased in the myocardium of diabetic mice, and the expression levels of necrotic apoptosis‐associated proteins, such as receptor‐interacting protein kinase (RIPK) 1/3 and NLRP3 inflammasome, were much higher in cardiomyocytes from SIRT3 KO diabetic mice than in wild‐type diabetic mice. In addition, elevated expression of pyroptosis and inflammation‐related proteins such as NLRP3, gasdermin D N‐terminal domain (GSDMD‐N), and interleukin‐1 beta (IL‐1β) was also found in high glucose‐induced H9C2 cells, and activation of SIRT3 inhibited high glucose‐mediated cellular pyroptosis [63]. Nonetheless, direct evidence for the exact mechanism by which the SIRT3 signaling pathway targets and regulates DCM cellular pyroptosis is not yet known, and more in‐depth studies are still needed.

#### 3.3.3 SIRT3 and Ferroptosis in the DCM

Ferroptosis is an iron‐dependent regulated form of cell death, distinct from programmed cell death such as necrosis, autophagy, and apoptosis [64]. In terms of biochemistry, ferroptosis is manifested by glutathione (GSH) depletion, glutathione peroxidase 4 (GPX4) inactivation, and an increase in divalent iron ions (Fe^2+^) and lipid peroxidation [65]. In recent years, many studies have confirmed the true existence of ferroptosis in DCM cardiomyocytes and its damaging effects on the heart [66, 67]. In the heart, SIRT3 has been shown to have a protective function that minimizes hypertrophy, fibrosis, and damage [7]. Recent studies [68] reported that SIRT3 may play a key role in ferroptosis, and inactivation of SIRT3 significantly enhanced GPX4‐mediated ROS stress and ferroptosis, which could be ameliorated by activation of SIRT3 in various types of diseases. SIRT3 is thought to protect the heart from oxidative stress and mitochondrial damage, inhibit ferroptosis, and ameliorate cardiac hypertrophy and fibrosis in DCM [9]. Baicalin improves DCM by regulating SIRT3 levels, restoring mitochondrial stability, and inhibiting ferroptosis in cardiomyocytes [69]. Thus, targeting SIRT3 to regulate ferroptosis is important for improving DCM.

### 3.4 SIRT3 and Endothelial Cell Dysfunction in DCM

The hyperglycemic, hyperlipidemic, inflammatory, and oxidative environment in DCM patients promotes the development of microvascular dysfunction. SIRT3 is involved in the regulation of vascular endothelial cell dysfunction in diabetic patients, mainly through the coordination of oxidative homeostasis metabolism and the control of ROS homeostasis [70, 71]. Studies have indicated that endothelial cells can be protected from high glucose‐induced mitochondrial dysfunction and apoptosis through the SIRT3/FOXO3 signaling pathway [72]. Furthermore, in high glucose‐induced human aortic endothelial cells, SIRT3 overexpression significantly improved peroxisome proliferator‐activated receptor α and endothelial‐type nitric oxide synthase (eNOS) expression and suppressed inducible nitric oxide synthase (iNOS) levels [73]. It has been found that SIRT3 KO endothelial cells reduced endothelial cell tube formation, migration, and aortic outgrowth, affecting angiogenesis and leading to myocardial capillary thinning and diastolic dysfunction [74]. In addition, Apelin, a bioactive peptide secreted by endothelial cells with potent angiogenic activity, promotes angiogenesis and participates in cardiac regulation [75]. In STZ‐induced diabetic mice, Apelin overexpression increases SIRT3 expression in mouse endothelial progenitor cells, improves vascular endothelial cell function, increases angiogenesis, and improves cardiac function in DCM, whereas these protections are lost in SIRT3 KO mice, suggesting that Apelin's improvement of vascular endothelial cell function is dependent on the presence of SIRT3 [76]. Similarly, Zeng et al. [37] found that endothelial SIRT3 may regulate cardiomyocyte glucose utilization and influence cardiac function. Specifically, SIRT3 KO disrupted glucose transport from endothelial cells to cardiomyocytes in a paracrine manner through Apelin to reduce cardiomyocyte glucose utilization, thereby affecting cardiac function. The above findings suggest that SIRT3 regulates endothelial cell function by affecting angiogenesis, cardiac regulatory protein secretion, and thereby ameliorates DCM myocardial injury.

## 4 Elevating SIRT3 to Improve DCM

**FIGURE 3:**
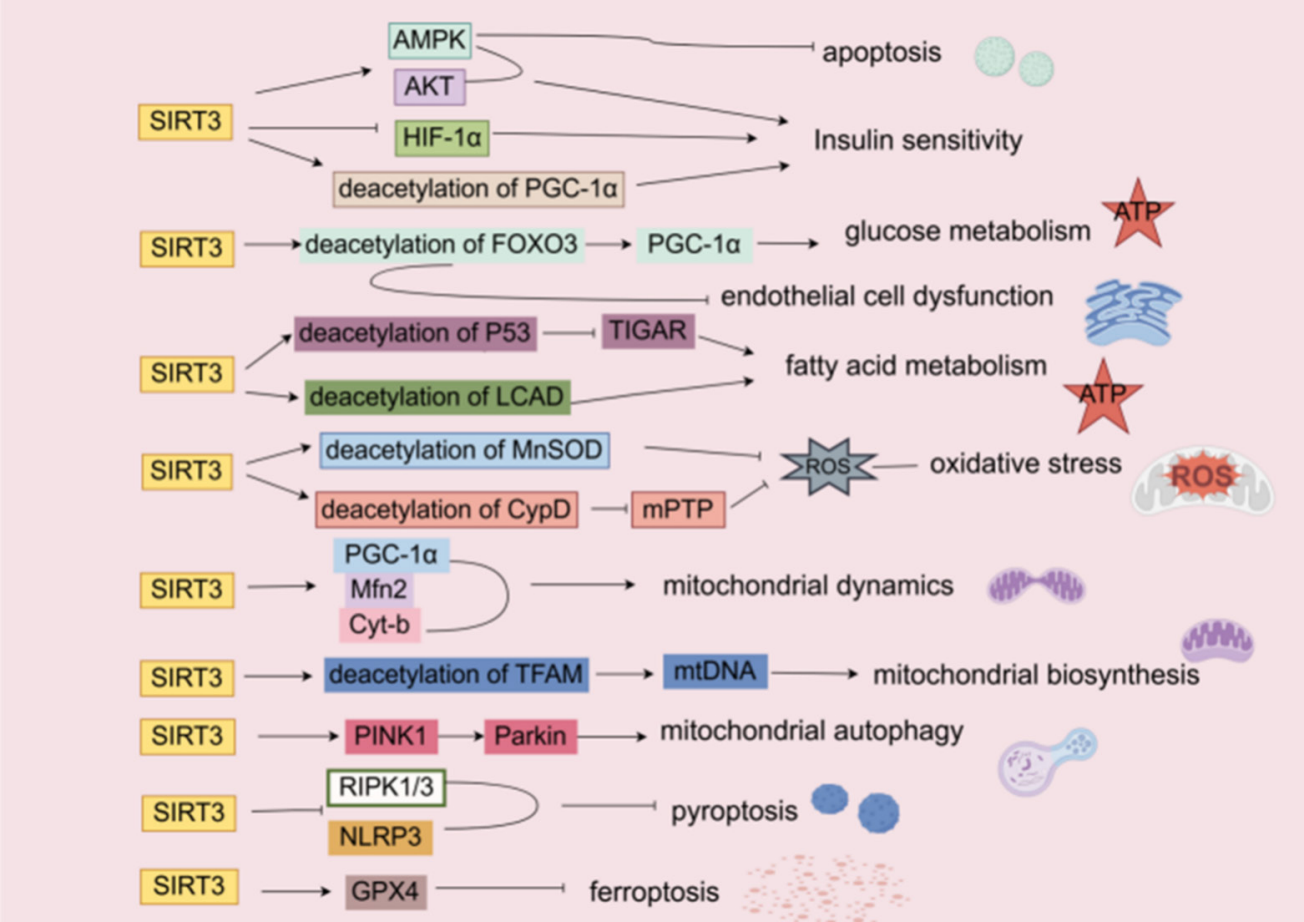
Mechanisms of SIRT3 in DCM. AMPK, AMP‐activated protein kinase; AKT, Ak strain transforming; HIF‐1α, Cyt‐b, Cytochrome b; CypD, Cyclophilin D; HIF‐1α, Hypoxia‐Inducible Factor 1‐α; PGC‐1α, Peroxisome Proliferator‐Activated Receptor Gamma Coactivator 1‐α; FOXO3, Forkhead box O 3; LCAD, GPX4, Glutathione Peroxidase 4; Long‐Chain Acyl‐CoA Dehydrogenase; Mfn2, Mitofusin 2; MnSOD, Manganese SuperOxide Dismutase (SOD2); mPTP, Mitochondrial Permeability Transition Pore; mtDNA, Mitochondrial DNA; NLRP3, nucleotide oligomerization domain like receptor protein 3; P53, Tumor protein p53; Parkin, Parkin Protein; PINK1, PTEN‐Induced putative Kinase 1; RIPK1/3, Receptor‐Interacting Protein Kinase 1/3; ROS, Reactive Oxygen Species; SIRT3, Silent information regulator 3 (sirtuin3); TFAM, Mitochondrial Transcription Factor A; TIGAR, TP53‐Induced Glycolysis and Apoptosis Regulator.

SIRT3 regulates cardiomyocyte energy metabolism, improves mitochondrial dysfunction, inhibits cardiomyocyte death, and ameliorates endothelial cell dysfunction through different mechanisms, and ultimately serves as a key player in improving DCM (Figure 3). So targeting SIRT3 may be a potential strategy for treating DCM, which can be ameliorated by enhancing SIRT3 activity and expression.

Lifestyle change is one of the important treatment modalities for cardiovascular disease, and changing an unhealthy lifestyle helps to regulate SIRT3 expression, which plays a role in the prevention and management of cardiovascular disease [77]. Studies have shown that diets high in sugar, fat, and salt all downregulate SIRT3 levels, leading to the development of cardiovascular disease; therefore, a “three lows” diet can minimize cardiovascular damage [78]. A study by Yu et al. [79] also showed that a certain degree of calorie restriction likewise contributes to the up‐regulation of SIRT3 expression in cardiomyocytes and improves cardiac function. In addition, short‐term exercise, especially intermittent aerobic exercise, can likewise promote SIRT3 expression and bring cardiovascular benefits [80]. In conclusion, lifestyle improvement can help elevate SIRT3 levels and play a role in alleviating cardiovascular disease, which is likewise important for improving DCM.

In terms of drug therapy, SIRT3 activation shows potential in improving DCM. It has been demonstrated that some western medications can ameliorate DCM by upregulating SIRT3. Melatonin attenuates cardiac remodeling and dysfunction in DCM by upregulating autophagy through activation of the MST1/SIRT3 signaling pathway, limiting apoptosis, and modulating mitochondrial integrity and biogenesis [51]. Metformin, a first‐line therapy for type 2 diabetes, can attenuate hyperglycemia‐induced atherosclerosis by upregulating SIRT3 expression [81]. Metformin activates the SIRT3‐AMPK axis, which can help to delay cardiac dysfunction associated with the metabolic syndrome [82], but further studies are needed to see whether metformin ameliorates DCM.

In addition, many studies have reported that the active ingredients of traditional Chinese compounds can improve DCM myocardial injury by targeting SIRT3 and elevating SIRT3 levels. Berberine improves cardiac insufficiency and cardiac hypertrophy in DCM mice by regulating SIRT3‐mediated lipid metabolism, promoting lipophagy, and reducing high glucose‐induced cellular lipotoxicity [83]. Baicalin inhibits the occurrence of ferroptosis and apoptosis in cardiomyocytes by activating the SENP1/SIRT3 signaling pathway, promoting the deacetylation of SIRT3, reducing the accumulation of ROS, and improving mitochondrial quality control [69]. Salidroside increased the expression of SIRT3 in cardiomyocytes, also promoted the translocation of SIRT3 from the cytoplasm to the mitochondria, and increased the deacetylation of mitochondrial proteins, such as MnSOD, which improved mitochondrial biogenesis and protected cardiac function in DCM mice [84]. Icariin may attenuate the development of DCM by preventing mitochondrial dysfunction through the Apelin/SIRT3 pathway, increasing SIRT3 expression [49]. Resveratrol activates SIRT3, regulates the acetylation status of TFAM, ameliorates mitochondrial dysfunction, and protects DCM cardiomyocytes [50, 55].

The experimental evidence above demonstrates that SIRT3 activation holds therapeutic promise in DCM, but most compounds lack specificity. For instance, melatonin and metformin have been shown to upregulate SIRT3 expression and activity, but they also influence other sirtuins and multiple signaling pathways. Natural compounds such as resveratrol and berberine exhibit pleiotropic effects, which complicate the attribution of their benefits solely to SIRT3 activation. Moreover, sustained SIRT3 overexpression has been associated with both tumor‐suppressive and tumor‐promoting effects in different cancers, raising concerns about the long‐term safety of SIRT3 activation. We have summarized known SIRT3 activators, their specificity, and current developmental status in Table 1.

**TABLE 1:** Summary of compounds that improve diabetes cardiomyopathy by targeting SIRT3.

Compound	Target/pathway	Specificity for SIRT3	Stage	Key outcomes	References
Melatonin	MST1/SIRT3	Moderate (also acts on SIRT1)	Preclinical	Anti‐apoptotic, improves mitochondrial function	[51]
Metformin	SIRT3‐AMPK	Low (broad metabolic effects)	Clinical (for diabetes)	Improve insulin resistance	[81, 82]
Berberine	SIRT3‐mediated lipophagy	Moderate	Preclinical	Reduces lipotoxicity, improves lipid metabolism	[83]
Baicalin	SENP1/SIRT3	Moderate	Preclinical	Inhibits ferroptosis and apoptosis	[69]
Salidroside	AMPK/Akt; PGC‐1α/TFAM; MnSOD	Moderate	Preclinical	Promotes the mitochondrial biogenesis	[84]
Icariin	Apelin/SIRT3	Moderate	Preclinical	Ameliorating mitochondrial dysfunction	[49]
Resveratrol	TFAM	Low (activates SIRT1, SIRT3)	Preclinical	Enhances mitophagy, reduces oxidative stress	[50, 55]

Abbreviations: AKT, Ak strain transforming; AMPK, AMP‐activated protein kinase; MnSOD, Manganese SuperOxide Dismutase (SOD2); MST1, mammalian Ste20‐Like kinase 1; PGC‐1α, peroxisome proliferator‐activated receptor gamma coactivator 1‐α; SENP1, SUMO/sentrin specific peptidase 1; SIRT3, silent information regulator 3 (sirtuin3); TFAM, mitochondrial transcription factor A.

Although extensive preclinical studies have established the protective role of SIRT3 in DCM, clinical evidence remains limited. A clinical study [85] examined myocardial tissue from obese and normal‐weight patients with left ventricular failure using Western blotting. It revealed a 46% reduction in SIRT3 levels in obese heart failure patients. Notably, the acetylation profile in obese patients' failing hearts was partially mediated by reduced SIRT3, which also correlated with elevated BNP levels and more severe ventricular dysfunction. This suggests that SIRT3 reduction mediates cardiac failure in obese patients, indicating that SIRT3 may be a potential target for mitigating ventricular dysfunction and slowing heart failure progression. However, challenges remain for SIRT3 as a biomarker: 1. Poor tissue specificity and limited clinical accessibility of myocardial biopsies make it controversial whether peripheral blood SIRT3 reflects myocardial activity; 2. Translational bottlenecks exist as existing SIRT3 agonists lack myocardial targeting. Future studies should validate the association between SIRT3 and DCM prognosis in multicenter prospective cohorts and develop myocardial‐targeted delivery systems.

## 5 Conclusions

Recent findings have demonstrated that SIRT3 plays an indispensable role in the occurrence and development of DCM. SIRT3 can delay the progression of DCM by regulating energy metabolism, improving mitochondrial dysfunction, inhibiting cardiomyocyte death, and improving endothelial cell function. Some western medications (melatonin, metformin) and active ingredients of traditional Chinese compounds (berberine, baicalin, salidroside, icariin, resveratrol) play a role in protecting DCM cardiomyocytes and improving cardiac function by activating the SIRT3‐related pathway and elevating the SIRT3 level. Therefore, SIRT3 agonists are expected to be new drugs for treating and delaying DCM [86]. However, the underlying mechanisms of the cardioprotective effects of SIRT3 are extremely complex and remain to be further elucidated. As far as clinical practice applications are concerned, the currently known SIRT3 agonists usually lack specificity, and they can also upregulate the expression of other sirtuins. SIRT2 and SIRT4 have been shown to have deleterious effects on ischemia–reperfusion injury and cardiac hypertrophy, respectively [87, 88]. SIRT3 agonists are conflicting in the treatment of different types of tumors [18, 89], and excessive SIRT3 activation may lead to tumorigenesis. Current research on SIRT3 primarily focuses on mitochondria, while studies on the function of cytoplasmic SIRT3 remain scarce. Future study should prioritize: (1) developing SIRT3‐specific small‐molecule agonists; (2) elucidating the role of SIRT3 subtypes in disease progression; (3) clinical validation of SIRT3 as a biomarker for DCM; (4) investigation of combination therapy using SIRT3 activators alongside conventional antidiabetic drugs. With sustained efforts, SIRT3‐based therapies may ultimately offer a novel strategy to combat the growing burden of DCM.

## Author Contributions

**Yuting Lin:** design, conceptualization, writing original draft. **Kun Yu:** assist in chart creation and literature search. **Jinjian Guo:** provided guidance on the design and implementation of the article. All authors read and approved the final manuscript. All authors have participated sufficiently in the work and agreed to be accountable for all aspects of the work.

## Funding

This study was supported by the National Natural Science Foundation (82574851), Fujian Natural Science Foundation (2024J01736).

## Conflicts of Interest

The authors declare no conflicts of interest.

## Data Availability

Data sharing not applicable to this article as no datasets were generated or analyzed during the current study.
